# Mitochondrial Dysfunction and Diabetes: Is Mitochondrial Transfer a Friend or Foe?

**DOI:** 10.3390/biology8020033

**Published:** 2019-05-11

**Authors:** Magdalene K Montgomery

**Affiliations:** Department of Physiology, School of Biomedical Sciences, University of Melbourne, Melbourne 3010, Australia; magdalene.montgomery@unimelb.edu.au; Tel.: +61-422-059-907

**Keywords:** mitochondrial dysfunction, insulin resistance, type 2 diabetes, mitochondrial transfer, exosomes

## Abstract

Obesity, insulin resistance and type 2 diabetes are accompanied by a variety of systemic and tissue-specific metabolic defects, including inflammation, oxidative and endoplasmic reticulum stress, lipotoxicity, and mitochondrial dysfunction. Over the past 30 years, association studies and genetic manipulations, as well as lifestyle and pharmacological invention studies, have reported contrasting findings on the presence or physiological importance of mitochondrial dysfunction in the context of obesity and insulin resistance. It is still unclear if targeting mitochondrial function is a feasible therapeutic approach for the treatment of insulin resistance and glucose homeostasis. Interestingly, recent studies suggest that intact mitochondria, mitochondrial DNA, or other mitochondrial factors (proteins, lipids, miRNA) are found in the circulation, and that metabolic tissues secrete exosomes containing mitochondrial cargo. While this phenomenon has been investigated primarily in the context of cancer and a variety of inflammatory states, little is known about the importance of exosomal mitochondrial transfer in obesity and diabetes. We will discuss recent evidence suggesting that (1) tissues with mitochondrial dysfunction shed their mitochondria within exosomes, and that these exosomes impair the recipient’s cell metabolic status, and that on the other hand, (2) physiologically healthy tissues can shed mitochondria to improve the metabolic status of recipient cells. In this context the determination of whether mitochondrial transfer in obesity and diabetes is a friend or foe requires further studies.

## 1. Introduction

The dysregulation of carbohydrate and lipid metabolism due to unbalanced diets (i.e., ‘fast food’) in western societies has led to a dramatic rise in the worldwide prevalence of obesity, with more than 1.9 billion adults worldwide considered overweight or obese in 2016 [1]. Undoubtedly, this number has increased even further within the last three years. Obesity is one of the most common causes of the development of insulin resistance, type 2 diabetes (T2D), and other related cardio-metabolic risk factors. As outlined by the National Institute for Diabetes and Digestive and Kidney Diseases (NIDDK), T2D is characterized by hyperglycaemia and is predominantly prevalent in middle-aged and older individuals (>45 years of age) [2]. Insulin resistance (i.e., pre-diabetes) and T2D are accompanied by a multitude of systemic and tissue-specific metabolic defects, including inflammation, oxidative and endoplasmic reticulum stress, and lipotoxicity (i.e ectopic lipid accumulation in peripheral tissues), as well as mitochondrial dysfunction [3]. Whether these metabolic aspects are a cause or consequence of insulin resistance remains a matter of debate, with studies pointing towards both scenarios, as reviewed in detail in [4,5,6]. This review will focus on mitochondrial dysfunction in T2D, with a special focus on the systemic implications of mitochondrial dysfunction occurring in peripheral metabolic tissues. Specifically, we will review evidence suggesting that dysfunctional mitochondria and/or mitochondrial DNA can be packaged into extracellular vesicles, secreted from the respective tissue and can impact other tissues in a paracrine or endocrine manner. 

## 2. Mitochondrial Function and Dysfunction

Mitochondria have a multitude of roles, with the most prominent one being the generation of ATP for the maintenance of cellular processes. In addition, mitochondria play a role in reactive oxygen species (ROS)-mediated signalling [7,8,9,10], apoptosis [11,12], calcium signalling [8,13], and haem [14,15] and steroid synthesis [16], just to name a few. Due to the importance of mitochondria in cellular energy metabolism, defects in the processes mentioned above have significant outcomes on tissue and systemic level. In this respect, the term ‘mitochondrial dysfunction’ was firstly mentioned in 1975 in the context of glucose intolerance [17]. While the term ‘mitochondrial dysfunction’ is nowadays commonly used in the scientific literature, the definitions and methods of assessment vary between studies. Mitochondrial dysfunction has been assessed as changes in gene expression of mitochondrial markers [18,19], protein content, or enzymatic activities of mitochondrial proteins [18,20], changes in mitochondrial size and shape [20,21], as well as functional assessment of mitochondrial oxidative capacity [22] and ROS generation [23]. For details on these studies and for mechanistic insights into possible causes of mitochondrial dysfunction, please refer to our recent review on ‘mitochondrial dysfunction and insulin resistance’ [24]. 

## 3. Mitochondrial Dysfunction in Obesity and Type 2 Diabetes

Since the first mention of mitochondrial dysfunction in the context of glucose intolerance in 1975 [17], the importance of mitochondrial function/dysfunction in insulin resistance has been disputed for many years. While some studies in both humans and rodents describe a decrease in mitochondrial function (decreased mitochondrial enzyme activities, decreased lipid metabolism, reduced mitochondrial size and number) in obese and insulin resistant individuals [25,26], others show no correlation [27,28] or even a compensatory increase in mitochondrial capacity with lipid oversupply [29,30], with most studies focusing on skeletal muscle (reviewed in detail in [24]). The discrepancies between studies in both rodents and humans suggest that mitochondrial dysfunction is not a requisite feature of insulin resistance and T2D, and that study outcomes (i.e., if mitochondrial dysfunction is present) are likely dependent on methodological approaches, the model system examined (human or rodent) and the definition of the term mitochondrial dysfunction in the context of each study. In addition, differences in mitochondrial defects between metabolic tissues need to be considered. While skeletal muscle is the most common tissue examined in the context of T2D and mitochondrial function [31], the importance of mitochondrial function in other tissues, such as heart, liver and pancreas, and the impact on systemic homeostasis, should not be underestimated. 

## 4. Tissue-Specific Effects of Mitochondrial Function and T2D 

Skeletal muscle is packed with mitochondria, which are required for ATP generation during muscle contraction. With the number of mitochondria in a cell being proportional to the energy demand of the cell, it is not surprising that skeletal muscle and heart are the two tissues with the highest mitochondrial abundance [32]. We have reviewed changes in mitochondrial function in skeletal muscle in the presence of insulin resistance and T2D in detail previously [24], with the major ‘conclusion’ being that skeletal muscle-related investigations into changes in mitochondrial function in the presence of insulin resistance or T2D have been ‘inconclusive’ (i.e., decreased, unchanged, or compensatory increased in mitochondrial function in insulin resistance have been suggested). Here we briefly describe changes in mitochondrial function and capacity in the heart, due to the hearts high rate of ATP production and turnover, and its reliance on mitochondrial metabolism to produce energy.

A large number of studies points towards mitochondrial defects being present in the heart in individuals with insulin resistance and diabetes [33,34,35,36,37] as well as in rodent models [38,39,40,41,42,43]. Markers examined in these studies included mitochondrial oxygen consumption, ROS and ATP generation, changes in mitochondrial size, and structure and calcium handling, as well as mRNA, protein, or enzymatic activity levels of markers of mitochondrial biogenesis, content, and oxidative capacity. Of note, whereas studies in humans are of an associative nature and limit investigations of cause-or-consequence scenarios, a majority of rodent models investigating cardiac function in the presence of diabetes use streptozotocin, a compound that shows high toxicity towards the pancreas and destruction of insulin-producing beta cells, and that is used for the generation of type 1 diabetes phenotypes [44]. The use of dietary interventions that more closely mimic human obesity and insulin resistance, as well as rapid improvements in generation and accessibility to transgenic rodent models are crucial for delineating cause-and-consequence relationships. For example, Turkieh and colleagues showed recently that apolipoprotein O (APOO) is overexpressed in diabetic hearts, and, using transgenic APOO mice, show that APOO links impaired mitochondrial function and the onset of cardiomyopathy [45]. Furthermore, using genetic approaches a recent study showed that myocardial function depends on balanced mitochondrial fusion and fission, and defined a critical regulator of cardiomyocyte survival, the mitochondrial metalloendopeptidase OMA1 [46]. In addition, expression of extracellular signal-regulated protein kinase 5 (Erk5) was shown to be lost in diabetic hearts, and cardiac-specific deletion of Erk5 in mice substantiated mitochondrial abnormalities and decreased fuel oxidation [47]. Lastly, epigenetic regulation of metabolic functions was shown to be important in the preservation of cardiac function. Specifically, an N-terminal proteolytically derived fragment of histone deacetylase 4 (HDAC4) was shown to be decreased in failing mouse hearts and that overexpression of this HDAC4 fragment improved calcium handling and contractile function [48]. 

While diving into the topic of cardiac dysfunction, I noticed that alterations in mitochondrial energy metabolism are a common feature of various forms of heart disease [49,50]. Most predominantly, diabetic cardiomyopathies (ventricular dysfunction in patients with diabetes mellitus) are associated with dysregulated oxidative substrate selection. In humans and rodents, obesity and insulin resistance are associated with increased myocardial fatty acid uptake and fatty acid oxidation [51,52], with a simultaneous decrease in glucose oxidation [53,54]. As fatty acid oxidation produces less ATP (per mol oxygen consumed), this scenario makes the heart less energy efficient [52]. In addition, increased fatty acid supply to the heart is also associated with oxidative stress [55] and accumulation of bioactive lipid intermediates that directly interfere with insulin signalling [56], and lead to a greater decline in heart function with age [57]. Of interest, therapeutic approaches to increase glucose utilization of the diabetic heart, either actively through activation of pyruvate dehydrogenase [58,59,60] or passively by inhibiting fatty acid oxidation [61,62], were shown to improve heart function.

## 5. Can Mitochondrial Dysfunction be Reversed?—Insight into Genetic and Pharmacological Interventions

Targeting mitochondrial oxidative capacity as a means to improve insulin sensitivity is questionable with contradicting findings in various genetic mouse models. For example, inhibition of mitochondrial fatty acid uptake (through inhibition of CPT1) improves insulin sensitivity in muscle of diet-induced obese mice, healthy male subjects and in primary human myotubes [63,64]. In addition, activating heat shock protein 72 (HSP72) in skeletal muscle was found to improve mitochondrial oxidative capacity and insulin sensitivity in the setting of lipotoxicity [65], whereas cardiac-specific overexpression showed no metabolic improvements [66]. Furthermore, increasing the capacity of fatty acid transport protein (FATP) 1 in skeletal muscle resulted in increased channelling of fatty acids into oxidative pathways, however without changes in muscle or systemic insulin sensitivity [67]. 

Similarly, contradicting findings come from lifestyle or pharmacological intervention studies. The major lifestyle interventions are diet and exercise, with both dietary restriction and physical activity shown to reduce the risk of developing insulin resistance and T2D [68,69], and to improve/restore muscle mitochondrial function [70,71]. While the impact of exercise on mitochondrial biogenesis and oxidative capacity is well accepted (as recently reviewed in [72]), the picture is not that clear in respect to mitochondrial improvements after dietary interventions. In addition to the studies above, other reports suggest that caloric restriction improves insulin sensitivity without changes (or even with decreases) in skeletal muscle mitochondrial oxidative capacity [73,74]. 

Although exercise and dietary interventions represent a good and some might say ‘easy’ way to improveinsulin sensitivity, long-term adherence to these lifestyle interventions is problematic and remains a public health challenge. In this respect, finding an ideal pharmacological agent that improves metabolic health, by improving mitochondrial function, has shown some promise in rodent models and in the clinic. One of the most well described modulators of oxidative metabolism are peroxisome proliferator-activated receptors (PPARs). For example, PPAR agonists were shown to increase mitochondrial content and oxidative capacity, in the livers of pre-diabetic mice [75], in adipose tissue [76] and skeletal muscle of insulin resistant and T2D patients [76,77] and mice [78], and these mitochondrial adaptations were accompanied by improvements in systemic insulin sensitivity. According to the NIH Clinial Trials register, PPAR agonists are now being trialled for cardiovascular disease, hypertension, pre-diabetes, and diabetes, and various PPAR agonists (e.g. pioglitazone, rosiglitazone, fibrates) have been FDA-approved for such therapeutic applications. In addition to PPAR ligands, a variety of other common anti-diabetic drugs have pronounced effects on mitochondrial function, with improvements in mitochondrial metabolism likely contributing to the systemic improvements in insulin sensitivity and glycaemic control [79]. While insulin therapy has been shown to improve mitochondrial oxidative phosphorylation [80] and ATP synthesis [81], SGLT2 inhibitors, which primarily act on inhibiting glucose reuptake in the kidney, also improve mitochondrial function in the heart [82,83,84] and in the brain [85]. However, the positive mitochondrial effects have not been supported by other studies, pointing towards potential inhibitory effects of SGLT2 inhibitors on the mitochondrial respiratory chain [86,87]. Furthermore, conflicting findings have also been reported for one of the most commonly prescribed anti-diabetic agents, the biguanide metformin. While some studies point towards metformin having beneficial effects on mitochondrial oxidative capacity [88] and mitochondrial fission [89], others show the opposite [90,91,92,93], with metformin being named ‘an energy disruptor’ [90]. Lastly, thiazolidinediones (TZDs) such as rosiglitazone have been shown to increase mitochondrial biogenesis [94,95]. For a detailed summary of the effects of anti-diabetic drugs on mitochondrial function, please refer to [79,96]. 

## 6. Intercellular Mitochondrial Transfer

In addition to ‘boosting’ mitochondrial oxidative capacity in the presence of mitochondrial dysfunction through genetic, lifestyle of pharmacological interventions, would it be possible to improve mitochondrial function in a target cell or tissue through mitochondrial transfer? Recent evidence suggests that intact mitochondria, mitochondrial DNA or other mitochondrial components (proteins, lipids or metabolites) can be found in the circulation [97,98,99,100] and can be transferred between cells or tissues [101,102,103]. Mitochondrial transfer between cells was shown in the context of leukaemia [101], acute lung injury [104], asthma [105], and acute respiratory distress syndrome (ARDS) [103], through the use of tunnelling nanotube-like structures between donor and recipient cells. Most importantly, recipient cells showed metabolic impairments which were improved upon transfer of functional mitochondria; e.g., acute myeloid leukaemia (AML) cells were less prone to mitochondrial depolarization after chemotherapy [101]. Other researchers turned to intravenous injections of intact mitochondria as a therapeutic approach for mitochondrial replenishment after cardiac injury [106]. While these studies highlight the protective effects of mitochondrial transfer in a variety of disease conditions, other studies point towards exogenous mitochondria having pro-inflammatory effects [107,108]. In the context of cancer, mitochondrial transfer from benign donor to recipient carcinoma cells elicited a chemo-resistant phenotype [109] and contributed to tumour proliferation [110]. Little is known about mitochondrial transfer in conditions of mitochondrial dysfunction and insulin resistance. Could transfer of intact mitochondria or mitochondrial DNA be a potential therapeutic means to improve mitochondrial dysfunction and subsequently insulin resistance? Could mitochondrial transfer on the other hand further worsen already substantiated metabolic defects? While intercellular transfer using nanotubes-like structures (as described above) is a potential transfer mechanism for cells in close proximity, this pathway is unlikely to ‘remodel’ whole organs and tissues, as would be required for example for skeletal muscle mitochondrial dysfunction. However, of interest, mitochondrial DNA and mitochondrial proteins have also been shown to be present in extracellular vesicles, particularly exosomes, and the significance of these findings will be discussed below.

## 7. Exosomes

**Figure 1:**
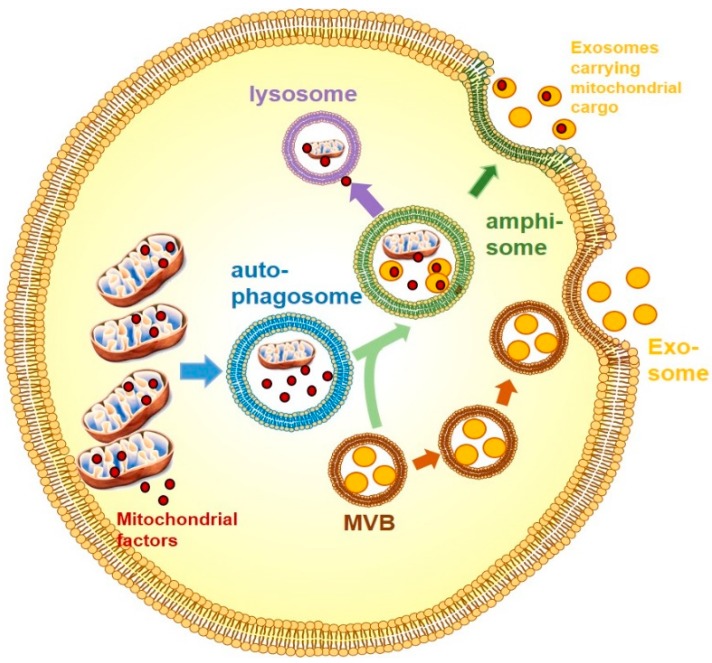
Exosomal secretion of intact mitochondria or mitochondrial factors. Mitochondria or mitochondrial components are packaged into autophagosomes for intracellular degradation. Autophagosomes can fuse with multi-vesicular bodies (MVBs) carrying exosomes, forming amphisomes. Amphisome contents can be either shuttled towards degradation in lysosomes or towards the cell surface where exosomes are released.

It has been known for more than 40 years that cells secrete vesicles during apoptosis [111]. More recent studies suggest that also healthy cells have the capacity to release vesicles into the extracellular environment, with exosomes having received substantial attention over the past couple of years, primarily due to early findings such as that dendritic cells secrete antigen-presenting exosomes [112] and that tumour cells can transfer exosomes to dendritic cells to subsequently induce potent antitumor effects [113]. The term exosome is used to distinguish extracellular vesicles with a diameter of 50 to 150 nm, that originate from the late endosomal pathway and are released in the extracellular space upon fusion of multivesicular bodies (MVBs) with the plasma membrane [114] (Figure 1). For detailed info on biogenesis of exosomes, mechanisms of exosome secretion and detailed information on cargo, please refer to a recent review on this topic [115]. Exosomes are lipid-bound vesicles that carry lipids, proteins and nucleic acids. Interestingly, up to 10 percent of exosomal proteins have been described as mitochondrial proteins [116]. However, it should be noted that the relative high number of mitochondrial proteins identified within exosomal preparations could be also due to "contamination" during the preparation procedure. Exosomes are released from a multitude of tissues and cell types, including metabolic tissues, such as skeletal muscle [117], liver [118] and adipose tissue [119,120]. The amount and cargo of released microvesicles differs in obesity and type 2 diabetes [121,122,123,124], with adipose-derived exosomes from obese individuals and mouse models being associated with insulin resistance [125,126] and plasma exosomes in obese, T2D individuals showing enrichment in molecules involved in inflammation and immune efficiency [127]. Also, plasma exosomes from obese rats, but not lean rats, induced significant oxidative stress and vascular cell adhesion protein 1 (VCAM-1) expression in endothelial cells, indicative of a pro-inflammatory vesicle phenotype [123].

Importantly, obesity-associated exosomes released from adipose tissue, macrophages or erythrocytes can transform healthy cells into metabolically defective cells, with robust effects on tissue-specific and systemic insulin sensitivity and overall glucose homeostasis [124,126,128]. Overall, it seems that extracellular vesicles reflect the diverse functional and dysfunctional states of the releasing cells, and it is therefore not surprising that specific vesicle/exosomal signatures are present in distinctive disease states, making exosomes useful biomarkers in the future [129].

## 8. Exosomes with Mitochondrial Cargo

As mentioned above, up to 10% of exosomal proteins are mitochondria-derived [116]. Initially, vesicles containing mitochondrial cargo were described as being transported within the cytosol towards peroxisomes (i.e., inter-organellar communication) [130], however other vesicle sub-types were also shown fuse with the late endosome or with multi-vesicular bodies for degradation [131], with selective enrichment of oxidized cargo [132]. Of interest, the stress-induced mitochondria-associated proteins Parkin and Pink1 are involved in the generation of these particular multi-vesicular bodies [133], highlighting increased vesicle generation upon autophagy-related stress signals, potentially as a means of mitochondrial quality control. While these studies suggest that these vesicle sub-types and mitochondrial components are destined for degradation, they can also be routed towards the cell surface and fuse with the plasma membrane [134] (Figure 1). It is unclear if increased mitophagy is associated with increased exosomal secretion of mitochondrial components, potentially as a means to get rid of oxidized proteins, in addition to lysosomal degradation.

## 9. Exosomes with Mitochondrial Cargo—Friend of Foe?

Exosomes containing increased content of mitochondrial lipids, proteins and nucleic acids were found to be released from adipose tissue of obese diabetic and obese non-diabetic rats [135], from pulmonary cells after cigarette smoke injury [136], and are found in plasma upon infection with the human T-lymphotropic retrovirus type 1 (HTLV-1) [99] and in circulating exosomes of breast cancer patients [137], highlighting the broad range of pathological states that are characterized by increased circulating exosomes carrying mitochondrial cargo. Some studies suggest that the cells and tissues releasing these particular exosomes show mitochondrial impairments. For example, it has been shown that mesenchymal stem cells target depolarized mitochondria to the plasma membrane, and that they similarly shed microRNA-containing exosomes, which have the capacity to inhibit macrophage activation, thereby de-sensitizing macrophages to the ingested mitochondria [138]. Cells might use exosomal secretion as a way of quality control, to restore cellular homeostasis and preserve cell viability, to some extent in the presence of mitochondrial dysfunction [139]. This would suggest that mitochondrial cargo within circulating exosomes is largely metabolically defective, and when fusing with recipient cells could either transfer these mitochondrial defects to the recipient cells, or could induce a physiologically adverse response. In support of this hypothesis, it has been shown that leukaemia cells, when challenged with chemotherapeutic drugs that induce oxidative stress, transfer their dysfunctional mitochondria to neighbouring bone marrow mesenchymal stem cells, leading to chemo-resistance [140]. In addition, exosomes can carry mitochondrial electron transport chain (ETC) complexes. Some of these ETC subunits are encoded by mitochondrial DNA. Translation within mitochondria occurs using a formylated initiating methionine (a mechanism still conserved from bacterial origins), and presence of extracellular formylated proteins can lead to an immune response and cytokine release [107]. 

While mitochondrial proteins are most commonly investigated in the context of exosomal transport, exosomes also carry lipids (a recent paper identified 1,961 lipid species in an exosomal and microvesicle screen [141]), with exosomes from hepatocellular carcinoma cells (Huh7) and human bone marrow-derived mesenchymal stem cells (MSC) specifically enriched in the mitochondrial inner membrane lipid cardiolipin [141]. This enrichment of cardiolipin, but exclusion of other lipid species, such as sphingolipids, within these particular exosomes suggests that cardiolipin might be actively sorted into exosomes of Huh7 cells and MSCs [141]. While the reason for this phenomenon is unknown, the authors suggest that the high concentration of cardiolipin might help in the stability and curvature of the vesicle membrane bilayer. On the other hand, increased secretion of cardiolipin (or other mitochondrial lipids) could impair mitochondrial function of the donor cells, as cardiolipin plays an important role in maintaining optimal mitochondrial function. In this respect, a loss of cardiolipin has been described in the diabetic heart [142,143,144] and during heart failure [145], while exercise leads to increased cardiolipin content in skeletal muscle [146]. If this is due to differences in synthesis, degradation or exosomal secretion requires further investigation. As mentioned above, exosomal secretion has been considered a way of quality control [139], with the donor cells ‘getting rid’ of damaged or oxidized cellular components. During conditions of increased mitochondrial ROS production and oxidative stress, as is the case in the presence of insulin resistance and T2D [147], polyunsaturated fatty acids in mitochondrial membranes (such as cardiolipins) are the primary targets of oxidative damage, which may lead to mitochondrial dysfunction [148]. While oxidized cardiolipin can be repaired enzymatically [149], exosomal export is also a likely pathway for clearance of oxidized cellular factors.

Looking at the other side of the coin, a recent study published in the journal *Blood* investigated the metabolic cross-talk between cancer cells and their microenvironment, and found that ‘healthy’ bone marrow stromal cells (BMSC) were made to transfer their mitochondria to neighbouring acute myeloid leukaemia (AML) cells, supporting the cancer cells’ growth [102]. An associated press release in Science Daily termed this phenomenon quite adequately as “Stealing from the body: How cancer recharges its batteries” [150]. A different study identified the complete mitochondrial genome within circulating extracellular vesicles from metastatic breast cancer patients, and showed that these extracellular vesicles can in turn transfer their mtDNA to cells with impaired metabolism, leading to restoration of metabolic activity [151]. The authors suggested that the transfer of mtDNA plays a role in mediating resistance to hormone therapy in these patients. 

**Figure 2:**
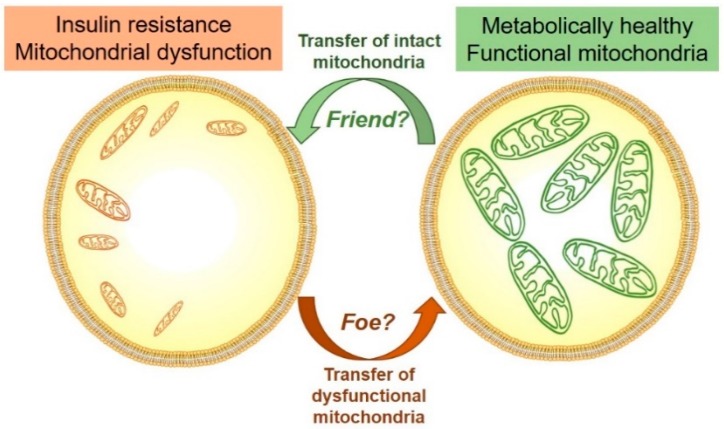
Is mitochondrial transfer in states of insulin resistance and mitochondrial dysfunction a friend or foe? In other pathological conditions, it has been shown that cells with mitochondrial impairments have the capacity to secrete mitochondrial cargo within exosomes that then impairs metabolic state of the recipient cells (i.e., foe). Other studies suggest that ‘healthy’ cells secrete mitochondrial cargo to improve the recipients cell metabolism (i.e., friend). Future studies will have to show if these phenomena are present states of mitochondrial dysfunction and insulin resistance.

It seems that, depending on the tissue/cell type and the pathological state examined, mitochondrial cargo can be either transferred from a cell with mitochondrial dysfunction to a cell with ‘healthy’ metabolic state, leading to metabolic deterioration of the recipient cells; or, on the other hand mitochondrial cargo can be transferred from a ‘healthy’ cell to a recipient cell with mitochondrial dysfunction, leading to the recipient’s metabolic improvement (Figure 2). The impact or physiological importance of exosomal transfer of mitochondrial cargo in the context of mitochondrial dysfunction in insulin resistant and T2D individuals is not known. Does skeletal muscle, heart or liver (or major metabolic tissues in general) have the capacity to shed mitochondria in the presence of mitochondrial dysfunction to rescue the donor’s cell energetic state? Could on the other hand mitochondrial cargo be transferred to metabolically deficient cells, to improve mitochondrial dysfunction in the recipient tissue (Figure 2)? 

## 10. Mitochondrial Dysfunction, T2D and Exosomal Transfer of Mitochondrial Cargo

Very little is known about exosomal transfer of mitochondrial cargo in the presence of mitochondrial dysfunction during the development of insulin resistance and T2D. It has been shown that in obese diabetic rats adipose-derived exosomes carry more mitochondrial lipids, proteins and nucleic acids [135]. Furthermore, lower circulating mtDNA content is associated with T2D [152] and severe proliferative diabetic retinopathy [153], with reduced peripheral blood mtDNA content potentially increasing the risk of impaired glucose-stimulated β cell function [152]. In addition, HbA1c, fasting plasma glucose and age of T2D onset are the major factors affecting mtDNA content [154]. While these studies assessed changes in mtDNA content in the circulation, this was not investigated in the context of exosomal transport. In a related matter, point mutations in the mitochondrial genome and decreases in mtDNA copy number have been linked to the pathogenesis of type 2 diabetes [155,156]. Compared with nuclear DNA repair, mtDNA repair mechanisms are significantly less efficient [157] and mtDNA is more susceptible to oxidative stress and mutations [158]. While the secretion of mtDNA within microvesicles has been described previously [159], little is known if mutated mtDNA can be secreted within exosomes and taken up by other cells.

Future studies will have to determine whether metabolic tissues, such as skeletal muscle or liver, have the capacity to shed defective mitochondrial components within exosomes, and if this process is affected in obese T2D individuals. Could on the other hand mitochondrial transfer be used as a means to improve mitochondrial defects (Figure 2)? It would be of interest to assess if targeted transfer of mitochondrial proteins or metabolites, or even fully functioning intact mitochondria, could improve mitochondrial dysfunction and have effects on insulin sensitivity and glucose homeostasis. 

## 11. Conclusions and Future Directions

Mitochondrial impairments have been described in the context of obesity, insulin resistance and T2D, in a variety of metabolic tissues. However, as studies reported opposing findings ranging from decreased mitochondrial function, to unaffected mitochondrial capacity, and even to an increase in mitochondrial oxidative metabolism in individuals and rodent models with insulin resistance or T2D, the requisite for the presence of mitochondrial dysfunction in these pathological states is unclear. In addition, genetic manipulations of mitochondrial proteins, as well as lifestyle and pharmacological interventions resulted in contradicting findings, and it is still unclear if targeting mitochondrial capacity is a useful therapeutic approach for the treatment of insulin resistance and glycaemic control. In the past years, it has become evident that metabolic tissues secrete microvesicles, such as exosomes, containing mitochondrial cargo, and that these exosomes have the capacity to fuse in a selective targeted manner with recipient cells and tissues, and influence the recipients’ cell metabolic status. Future studies will have to show if tissues with mitochondrial impairments in states of obesity and T2D have the capacity to ‘shed’ their dysfunctional mitochondria to improve their metabolic status, or if dysfunctional tissues have the capacity to ‘ingest’ exosomes with mitochondrial cargo to improve their oxidative metabolism.
